# Recent advances in faba bean genetic and genomic tools for crop improvement

**DOI:** 10.1002/leg3.75

**Published:** 2021-02-18

**Authors:** Hamid Khazaei, Donal M. O'Sullivan, Frederick L. Stoddard, Kedar N. Adhikari, Jeffrey G. Paull, Alan H. Schulman, Stig U. Andersen, Albert Vandenberg

**Affiliations:** ^1^ Department of Plant Sciences University of Saskatchewan Saskatoon Saskatchewan Canada; ^2^ School of Agriculture, Policy and Development University of Reading Reading UK; ^3^ Department of Agricultural Sciences, Viikki Plant Science Centre, and Helsinki Sustainability Science Centre University of Helsinki Helsinki Finland; ^4^ Plant Breeding Institute, Faculty of Science The University of Sydney Narrabri New South Wales Australia; ^5^ School of Agriculture, Food and Wine The University of Adelaide Adelaide South Australia Australia; ^6^ Production Systems Natural Resources Institute Finland (Luke) Helsinki Finland; ^7^ Institute of Biotechnology and Viikki Plant Science Centre University of Helsinki Helsinki Finland; ^8^ Department of Molecular Biology and Genetics Aarhus University Aarhus Denmark

**Keywords:** breeding, gene discovery, genomic resources, mapping population, *Vicia faba*

## Abstract

Faba bean ( *Vicia faba* L.), a member of the Fabaceae family, is one of the important food legumes cultivated in cool temperate regions. It holds great importance for human consumption and livestock feed because of its high protein content, dietary fibre, and nutritional value. Major faba bean breeding challenges include its mixed breeding system, unknown wild progenitor, and genome size of ~13 Gb, which is the largest among diploid field crops. The key breeding objectives in faba bean include improved resistance to biotic and abiotic stress and enhanced seed quality traits. Regarding quality traits, major progress on reduction of vicine‐convicine and seed coat tannins, the main anti‐nutritional factors limiting faba bean seed usage, have been recently achieved through gene discovery. Genomic resources are relatively less advanced compared with other grain legume species, but significant improvements are underway due to a recent increase in research activities. A number of bi‐parental populations have been constructed and mapped for targeted traits in the last decade. Faba bean now benefits from saturated synteny‐based genetic maps, along with next‐generation sequencing and high‐throughput genotyping technologies that are paving the way for marker‐assisted selection. Developing a reference genome, and ultimately a pan‐genome, will provide a foundational resource for molecular breeding. In this review, we cover the recent development and deployment of genomic tools for faba bean breeding.

## 1 INTRODUCTION

Faba bean ( *Vicia faba* L.) is one of the first domesticated food legumes and has a long history of cultivation; seeds as old as 14,000 years were identified in the southern Levant (Caracuta et al., 2016). Faba beans are widely grown for food and feed as a generous source of high‐quality protein, dietary fibre and other valuable nutrients (Duc, 1997; Khazaei & Vandenberg, 2020). The protein content of faba bean seeds is about 29% of the dry matter (Warsame et al., 2018), which makes it one of the main sources of affordable protein for people in the Middle East, Latin America and Africa, and for livestock feed in many developed countries. Faba bean, like most other legumes, forms a symbiosis with nodule‐forming bacteria that have nitrogen fixing ability, which provides major benefits to cropping systems and the environment and contributes to agricultural sustainability by soil improvement. It is considered an excellent protein crop due to its ability to provide nitrogen inputs into temperate agricultural systems on account of its wide adaptation (Rispail et al., 2010), as well as its high yield potential and nitrogen‐fixing capacity even when nitrogen is present in the soil (Herridge et al., 2008), compared with other grain legumes (Cernay et al., 2015). These particular nitrogen‐fixing traits in combination with yield potential mean that faba bean can be produced in a sustainable manner, making it particularly well‐suited for providing the protein required for the globally expanding plant‐based food chain. Faba bean delivers plant protein products suitable for consumption both by those with soybean ( *Glycine max* (L.) Merr.) allergy or intolerance and by those wishing local products. According to Food and Agriculture Organization Corporate Statistical Database (2019), faba bean is the fourth most widely grown cool‐season grain legume (pulse) globally after pea ( *Pisum sativum* L.), chickpea ( *Cicer arietinum* L.), and lentil ( *Lens culinaris* Medik.), with annual production of around 4.5 million tonnes from nearly 2.5 Mha.

Faba bean improvement is currently impeded by development of rich genomic resources having not kept pace with those of other cool‐season grain legumes. Faba bean is a partially allogamous diploid species with six pairs of remarkably large chromosomes. Its genome is one of the largest of any diploid field crop, about 13 Gbp in the haploid complement (Soltis et al., 2003) and contains more than 85% repetitive DNA (Novák et al., 2020). The large genome of faba bean is 2.9, 3.0 and 15.9 times larger than pea, lentil and chickpea, respectively. Assembly of the faba bean genome and map‐based cloning was delayed both due to its genome complexity (e.g. abundance of transposable elements) and the lower investment in its study compared with, for example, soybean. In the absence of a reference genome assembly for this species, high‐throughput approaches such as transcriptome analysis have been efficient tools for enrichment of genomic resources (e.g. Arun‐Chinnappa & McCurdy, 2015; Braich et al., 2017; Khan et al., 2019; Ocaña et al., 2015; Ray et al., 2015). However, from these reported transcriptome datasets, only limited DNA sequence data are available in public databases (Mokhtar et al., 2020). Additionally, the development of high‐density genetic maps derived from multiple populations and gene‐based molecular markers, particularly those developed by Webb et al. (2016) and Carrillo‐Perdomo et al. (2020), has paved the road to marker‐assisted selection (MAS) and gene discovery. For example, the elucidation of the biosynthetic pathway for the pyrimidine glycosides vicine and convicine (v‐c) (Björnsdotter et al., 2020), which have been the main factors limiting faba bean cultivation and usage in many warm regions, was not possible without the combination of transcriptome data (Ray et al., 2015) and gene‐based comparative mapping approaches (Khazaei et al., 2015, 2017).

Two recent review papers on this topic cover the coming of age of faba bean genetics and genomics in some detail (see Maalouf et al., 2019; O'Sullivan & Angra, 2016), but, since then, major progress on the key seed anti‐nutrients v‐c (Björnsdotter et al., 2020), seed coat tannins (e.g. Gutiérrez et al., 2020; Gutiérrez & Torres, 2019), as well as improved mapping approaches (Carrillo‐Perdomo et al., 2020) and transcriptome data (see Section 3), has been made (e.g. Gao et al., 2020; Wu et al., 2020; Yang et al., 2020). We provide here a comprehensive review on the mapping population and genomic resources in this species.

## 2 GENOMIC RESOURCES

### 2.1 Genetic maps

Genetic linkage maps have been developed in faba bean using different types of populations and molecular markers (Table 1). Sirks (1931) was the first to report a faba bean genetic map, identifying 19 genetic factors that formed four linkage groups. His genetic resources were lost during World War II. Four decades later, Sjödin (1971) used translocation lines for the assignment of different loci (for morphological observations, flower and seed coat colour) to their respective chromosomes. Genetic mapping studies were developed in the 1990s first with the aid of morphological markers, isozymes, seed protein genes and random amplified polymorphic DNA (RAPD) markers. Later, the development of expressed sequence tags (ESTs), microsatellites or single sequence repeats (SSRs), EST‐SSRs and single nucleotide polymorphism (SNP) markers helped to enrich faba bean genetic studies and breeding. The first DNA‐based linkage map in faba bean was constructed with only 17 markers, of which 10 were RFLPs (restriction fragment length polymorphism) (van de Ven et al., 1991). The first set of SSR markers were developed by Požárková et al. (2002) and then mapped by Román et al. (2004). A composite gene‐based map, anchored with orthologous markers mapped in *Medicago truncatula* Gaertn., was developed by Ellwood et al. (2008); synteny and genic collinearity among the legumes make the data applicable to *V. faba* and other legumes (Lee et al., 2017). Kaur, Kimber, et al. (2014) reported the first exclusively SNP‐based generic map of faba bean. Satovic et al. (2013) reported the first reference consensus genetic map, which covered 4062 cM (centiMorgan) in six main linkage groups, corresponding to the six chromosomes of faba bean. Table 1 shows that with the development of faba bean sequences and marker datasets, there was a correspondingly encouraging increase in the density and utility of gene‐based genetic maps. In the last few years, the significant advancements in genotyping and sequencing technologies have led to two new SNP‐based highly dense consensus maps. An international effort resulted in the first consensus map for six mapping populations, based on SNP markers derived from *M. truncatula* (Webb et al., 2016). It contained 687 SNP markers on six linkage groups, each presumed to correspond to one of the faba bean chromosomes. Carrillo‐Perdomo et al. (2020) recently reported the most saturated consensus genetic map to date: it was constructed using three mapping populations and encompassed 1728 SNP markers distributed in six linkage groups. Solid proof of macro‐synteny was also observed between this map and the most closely related legume species that have been sequenced. Recently, a database of ESTs, EST‐SSRs, mtSSRs (mitochondrial‐simple sequence repeats) and microRNA‐target markers in faba bean has been launched (Mokhtar et al., 2020). Now that most pulse genomes are available, it is important to implement comparative genomic approaches, which will ultimately assist in the identification of candidate genes, quantitative trait loci (QTL) mapping, and assembly of the genome in faba bean.

**TABLE 1:** Information on available genetic maps constructed from bi‐parental population and traits mapped in faba bean

Population	Marker type	Population type	Population size	Map length (cM)	Ave. inter‐marker distance (cM)	Mapped traits	References
172 × Optica	7 RFLPs, 4 morphologicals, 3 isozymes, 3 RAPDs	BC		231			van de Ven et al. (1991)
Vf6 × (Vf173, Vf35)	43 RAPDs, 7 isozymes, 1 RFLP	2 F_2_s	20 + 44	300–350			Torres et al. (1993)
172 × Optica	8 morphologicals, 7 RFLPs, 4 isozymes, 4 RAPDs	BCF_2_		300		Biochemical and morphological traits	Ramsay et al. (1995)
Vf6 × (Vf2 T5,6;^a^ Vf33 T3,4; Vf159 T4,5,6)	147 RAPDs, 9 isozymes, 1 morphological	7 F_2_s	813 (total)	850			Satovic et al. (1996)
Vf6 × (Vf17, Vf27, Vf46)	105 RAPDs, 7 isozymes, 3 seed protein genes, 1 morphological	3 F_2_s	175	1200	20	Seed weight	Vaz Patto et al. (1999)
34Morocco × Kristall 25	77 RAPDs	F_7_	57	973	14.66		Surahman (2001)
Vf6 × Vf136	117 RAPDs, 2 isozymes, 2 seed protein genes	F_2_	196	1445	13.72	Broomrape and ascochyta blight resistance	Román et al. (2002; 2003)
Vf6 × (Vf2 T5,6; Vf33 T3,4; Vf27; Vf27 T4,6; Vf136; Vf159 T4,5,6)	176 RAPDs, 6 isozymes, 4 SSRs, 3 seed protein genes, 2 morphologicals	11 F_2_s	654 (total)	1559	8	Rust, broomrape and ascochyta blight resistance	Román et al. (2004)
29H × Vf136	94 RAPDs, 4 isozymes, 3 SSRs, 2 seed protein genes	F_2_	159	1308		Rust and ascochyta blight resistance, and agronomic traits	Avila et al. (2003; 2004; 2005)
Vf6 × Vf27	151 ITAPs	F_6_	94	1686	14.6		Ellwood et al. (2008)
Côte D'Or/1 × BPL 4628	131 RAPDs, 1 morphological	F_6_	101	1635	14.73	Frost tolerance and physiologically related traits	Arbaoui et al. (2008)
Vf6 × Vf136	238 RAPDs, 21 ISMs, 6 SSRs, 5 EST‐derived markers, 4 isozymes, 2 STSs, 1 SCAR	F_6_	165	2857	12.72	Ascochyta blight and broomrape resistance	Díaz‐Ruiz, Satovic, et al. (2009), Díaz‐Ruiz, Torres, et al. (2009) and Díaz‐Ruiz et al. (2010)
Vf6 × Vf27	167 EST‐derived markers, 71 RAPDs, 11 SSRs, 3 RGAs, 3 seed protein genes, 2 isozymes, 1 morphological	F_6_	124	1875	7.26	Flowering, yield‐related traits, plant architecture and yield	Cruz‐Izquierdo et al. (2012) and Ávila et al. (2017)
91825 × K1563	128 SSRs	F_2_	129	1587	12.4		Ma et al. (2013)
29H × Vf136	121 RAPDs, 38 EST‐derived markers, 6 SSRs, 5 RGAs, 1 defense‐related gene, 1 seed protein gene	F_7:8_	119	1402	9.87	Broomrape resistance	Gutiérrez et al. (2013)
Vf6 × Vf27, Vf6 × Vf136, 29H × Vf136	729 markers in total	3 RILs	124 + 165 + 119	4613	6	Consensus map	Satovic et al. (2013)
Icarus × Ascot	465 SNP markers, 57 EST‐SSRs	F_5:6_	95	1217	2.3	Ascochyta blight resistance and flowering time	Kaur, Kimber, et al. (2014) and Catt et al. (2017)
Mélodie/2 × ILB 938/2	188 SNP markers, 1 morphological	F_5_	211	928	5.8	Drought adaptation‐related and morphological traits, and vicine‐convicine	Khazaei et al. (2014a, 2014b, 2015)
Nubaria 2 × Misr 3	552 EST‐SSRs	F_2_	109	688	1.25		El‐Rodeny et al. (2014)
Albus × BPL 10, Albus × 29H, Hedin × CGN07715 cf‐3, NV644–1 × IG 12658, Mélodie/2 × ILB 938/2, Côte D'Or/1 × BPL 4628/1521	687 SNP markers	4 F_2_s, 2 RILs	136 + 165 + 52 + 192 + 200 + 101	1404	2.6	Consensus map, flower color (*zt1*)	Webb et al. (2016)
Fiord × Doza#12034	2784 SNP markers	F_6_	104	1027	0.37	Rust resistance	Ijaz (2018)
91825 × K1563	465 SSRs	F_2_	129	4517	9.71		Yang et al. (2019)
Nura × Farah	1152 SNP markers	F_4_	145	1022	1.45	Ascochyta blight resistance	Sudheesh et al. (2019)
Disco/2 × ILB 938/2	257 SNP markers, 2 morphologicals	F_6_	176	918	5.4	Flower color (*zt2*)	Zanotto et al. (2020)
(Nova Gradiska, Silian & Quasar) × Hiverna	1728 SNP markers	3 F_3_s	102 + 147 + 96	1548	0.89	Consensus map	Carrillo‐Perdomo et al. (2020)
Vf6 × Vf27	Cruz‐Izquierdo et al. (2012) + 44 KASPs and 37 dehiscence‐related markers	F_8:9_	124	4421		Pod dehiscence	Aguilar‐Benitez et al. (2020)

Abbreviations: EST, expressed sequence tags; ISM, intron‐spanning marker; ITAP, intron targeted amplified polymorphism; KASP, kompetitive allele specific PCR; RAPD, random amplified polymorphic DNA; RFLP, restriction fragment length polymorphism; RGA, resistant gene analogs; RIL, recombinant inbred lines; SCAR, sequence characterized amplified region; SNP, single nucleotide polymorphism; SSR, simple sequence repeat; STS, sequence tagged sites.

^a^ T refers to the assignment of linkage groups to chromosomes by trisomic segregation.

### 2.2 Mapping populations

Published studies in faba bean to date have mostly involved bi‐parental populations, derived from crosses between two inbred lines. Several types of bi‐parental mapping populations, such as F_2_, backcrosses and recombinant inbred lines (RILs), have been employed for genetic map construction and trait mapping. The relatively large set of interconnected bi‐parental populations that segregate for diverse important traits in this species will help advance faba bean breeding (Table 1). These types of populations are easy to construct and represent a powerful tool for QTL detection. Their optimal allele frequency and low rate of linkage disequilibrium decay within chromosomes means that only a few hundred RILs/markers are needed to map a QTL (Scott et al., 2020). Despite the advantages of bi‐parental populations, their mapping precision is low due to the low total amount of genetic recombination, as only two alleles are present at any locus, and to the low amount of genetic diversity that can be created by only two founders. These factors may limit the number of QTLs captured. Multi‐parent populations have been developed to cope with the limitations of bi‐parental populations (Scott et al., 2020). In faba bean, a multi‐parent population derived from 11 European winter bean founders was created and employed to identify genomic regions controlling frost adaptation (Sallam & Martsch, 2015). A multi‐parent population from four founders (ILB 938/2, Disco/2, IG 114476 and IG 132238) was developed for preliminary characterization of important morphological and biochemical traits (Khazaei, Stoddard, et al., 2018). A genetic map with 11 K loci is being developed using a 50 K Axiom SNP genotyping array (O'Sullivan et al., 2019). This population segregates for a number of traits including v‐c, seed coat tannin (white‐flowered parent carrying the *zt2* gene), seed size and colour, and branching. A MAGIC (multi‐parent advanced‐generation intercross) population comprising over 2000 F_4_ individuals is currently under development at ICARDA (International Center for Agricultural Research in Dry Areas), combining eight diverse parents with sources for heat, drought, ascochyta blight, chocolate spot, rust and broomrape resistance (Maalouf et al., 2019). Because in multi‐parental populations there can be as many alleles per locus as founders, quantifying genetic interactions between loci requires the large numbers of individuals (>1000), found in typical MAGIC populations.

Table 2 lists the faba bean genotypes and parental lines that have been used for genetic map construction or transcriptome analysis. Over 70% of the germplasm used for mapping purposes belongs to the Mediterranean adaptation zones (Australia, southern Europe and North Africa). The global collection of faba bean germplasm across 37 genebanks exceeds 43,000 accessions. The ICARDA collection comprises more than 8500 accessions held in Lebanon and Morocco by April 2020 (20% of the global collection, Westengen et al., 2020). Despite the wealth of faba bean germplasm, characterization and preliminary evaluation remain a challenge. Faba bean is represented in the collections by only the cultivated forms, and a wide range of variation in plant and seed phenotypic characteristics have been reported (Khazaei, 2014; Maalouf et al., 2019). The development of a reference genome, gene functional analyses and genotype–phenotype association, together with the development of high‐throughput genotyping platforms, will facilitate characterization of the genetic diversity within the germplasm collections as well as understanding of its potential. It will aid exploitation of the diversity as a key resource for breeding.

**TABLE 2:** Information on faba bean germplasm used for genetic population construction and transcriptome analysis purposes

Line	Origin/donor	Trait(s) of interest	Description
**Mapping population**			
Ac1655	Australia	Rust resistance	European line (V‐300) introduced from Spain (Adhikari et al., 2016)
Albus^a^	Poland	Low tannin	White‐flowered (*zt1*), Albus (Latin) means white
Ascot^a^	Australia	Resistant to ascochyta blight	Selection from cv. Fiord. Original source of germplasm is Greece (Kaur, Cogan, et al., 2014)
BPL 10^a^	Jordan	Nematode resistant	Pure line selection from accession IG 101769 (ILB 6)
BPL 228 (34Morocco)	Morocco		Pure line selection from IG 11335 (ILB 141)
BPL 4628	China	Frost tolerant	Pure line selection from IG 106387 (ILB 3009) from Anhui, China
CGN07715	GAUG, Germany	Closed flower	From CGN grain legumes collection, Wageningen, Netherlands
Côte d'Or	INRA, France	Frost tolerant	Old French winter bean from Côte d'Or region of Burgundy (Picard et al., 1985). Yellow (buff) seed coat (*Yg*)
Disco	INRA, France	Low tannin	Low v‐c, white‐flowered (*zt2*)
Doza^a^	Australia	Rust resistance	Pedigree: Ac383 × triple White. Original sources of germplasm are Ethiopia and Sudan, respectively
Farah^a^	Australia	Resistant to ascochyta blight	Selection from cv. Fiesta (selection from BPL 1196 from Spain) ( Kaur, Cogan, et al., 2014 )
Fiord^a^	Australia		The first faba bean cultivar released in Australia. Selection from Ac59 from the island of Naxos, Greece (Kaur, Cogan, et al., 2014)
Hedin^a^	GAUG, Germany	Highly inbred and autofertile, small seed size, and high seed number	It has already been adopted in a number of genomics projects as a reference genotype. Released in 1986 and has “Herz Freya” in its background
Hiverna	Germany	Frost tolerant	Large‐seeded winter bean, from NPZ released in 1986 (Link et al., 2010)
ILB 938 (IG 12132)	Andean region of Colombia and Ecuador	Drought adaptation, chocolate spot and rust resistance	ILB 938 (BPL 1179) is the result of mass selection from ILB 438 (BPL 710) based on seed size (Khazaei, Link, 2018). It carries a rare allele (*ssp1*) that decouples pigmentation in flowers from that in stipules ( Khazaei et al., 2014b )
Icarus^a^	Australia	Resistant to chocolate spot and rust	Icarus was derived from BPL 710 (see above) ( Kaur, Cogan, et al., 2014 )
IG 12658	Ethiopia	Dwarf	A dwarf accession carrying gibberellic acid deficiency gene (Hughes et al., 2020)
K1563	China	Winter bean	Small‐seeded
Kasztelan	Poland	Low tannin	White‐flowered (*zt1*), the NIAB accession code is NV644
Kristall 25	Germany		Developed in Lochow Petkus in 1973
Mélodie	INRA, France	Low v‐c	High water use efficiency (Khazaei et al., 2014a)
Misr 3	Egypt	Resistance to broomrape	Early flowering, small‐seeded. Pedigree: ((Giza 3 × ILB 938) × Cairo 241)) × (Giza 3 × 23A/45/76) (Attia et al., 2013)
Nova Gradiska	Croatia	Resistance to seed weevils (*Bruchus* spp.) (Carrillo‐Perdomo et al., 2019)	Small‐seeded
Nubaria 2	Egypt	Drought adaptation	Adapted to the Nubaria region in Egypt. Late flowering, large‐seeded. Pedigree: ILB 1550 × Radiation 2095/76
Nura	Australia	Resistant to ascochyta blight and moderate resistant to chocolate spot	Pedigree: Icarus × Ascot. Original sources of germplasm are Ecuador and Greece, respectively ( Kaur, Cogan, et al., 2014 )
Optica	Netherlands	Resistant to freezing, low tannin	Large‐seeded, white‐flowered (*zt1*)
Quasar	UK	Resistance to seed weevils (Carrillo‐Perdomo et al., 2019)	Winter bean adapted to oceanic climate
Silian	Northern Sudan		Small‐seeded
Vf6	IFAPA, Spain	Resistant to ascochyta blight	Asynaptic breeding line program from Córdoba
Vf27	IFAPA, Spain	Pod dehiscent	*Paucijuga* type
Vf136^a^	IFAPA, Spain	Moderate level of resistance to broomrape	From the progeny selection of Vf1071 × alameda. Vf1071 is a broomrape resistant line selected from cv. Giza 402. Alameda is a commercial variety well adapted to southern Spain
172	Afghanistan	High levels of post‐harvest seed dormancy	*Paucijuga* type
91825	China	Winter bean	Large‐seeded
29H^a^	INRA, France	Resistant to ascochyta blight	Small‐seeded breeding line developed at INRA
**Transcriptome**			
AO 1155	INRA, France	Low v‐c	Small‐seeded, white‐flowered (*zt1*)
CDC Fatima	Canada		An established cultivar developed for use in the prairie provinces of Canada. Selection from a landrace known as Chinese broad bean (Graf & Rowland, 1987)
Hassawi‐2	Saudi Arabia	Drought adaptation	Local landrace
SSNS‐1	Canada	Small‐seeded	Bulk selection from cv. Ackerperle from Germany
Tongxian‐2	China	Winter bean	Vegetable type
Windsor	UK		Large‐seeded, long pods
Wizard	UK	High‐yielding with large attractive seeds, ascochyta blight resistance	Large‐seeded winter bean from Wherry & Sons, UK, released in 2002
Y078	China	Salt sensitive	
Y134	China	Salt tolerant	

*Note*: ICARDA maintains faba bean germplasm in two classes, international legume bean (ILB) accessions from different countries, and bean pure line (BPL) accessions that are derived through selfing from accessions drawn from the ILB collection.

Abbreviations: CDC, Crop Development Centre; CGN, Centre for Genetic Resources, the Netherlands; GAUG, Georg‐August‐University, Göttingen; ICARDA, International Center for Agricultural Research in Dry Areas; IFAPA, Instituto de Investigación y Formación Agroalimentaria; INRA, Institut National de la Récherche Agronomique; NAIB, National Institute of Agricultural Botany; v‐c, vicine‐convicine.

^a^ Used for both mapping and transcriptome research.

### 2.3 Trait mapping

The first faba bean QTL mapping study was reported by Ramsay et al. (1995), who detected several loci for morphological and biochemical traits including v‐c. QTL mapping in faba bean for biotic stresses, such as resistance to pathogenic fungi or parasitic plants, has been attempted (Table 1). Two of the major constraints in Mediterranean climates, namely, ascochyta blight (caused by *Ascochyta fabae* Speg.,) and broomrape ( *Orobanche crenata* Forsk. and *O. foetida* Poir.), have been widely subjected to QTL studies using F_2_ and RIL populations (Table 1). The QTLs accounting for significant proportions of ascochyta blight resistance have been validated in multi‐environment trials (Atienza et al., 2016). In addition, some attention has been given to rust resistance (*Uromyces viciae‐fabae* (Pers.) J. Shört.) (Avila et al., 2003; Ijaz, 2018). Recently, two mapping populations (Fiord × Doza#12034 and Fiord × Ac1655) have been developed at the University of Sydney, in which KASP (Kompetitive Allele Specific PCR) markers for rust resistance genes *Uvf‐2* and *Uvf‐3* have been identified (Ijaz, 2018). However, until now, there has been no attempt to map QTLs or genes governing chocolate spot (caused by *Botrytis fabae* Sard.) resistance, in spite of the importance and widespread nature of this disease globally. A few RIL populations suitable for chocolate spot genetic studies have been developed using ILB 938, an accession with proven resistance to chocolate spot (reviewed by Khazaei, Link, et al., 2018). Two mapping populations (Mélodie/2 × ILB 938/2 and Disco/2 × ILB 938/2) have been phenotyped at the University of Saskatchewan and QTL mapping is underway. In addition, a list of faba bean accessions with resistance to chocolate spot is available (Maalouf et al., 2016).

Some progress has been made in identifying QTLs for abiotic stresses such as frost tolerance (Arbaoui et al., 2008; Sallam et al., 2016; Sallam & Martsch, 2015), traits related to drought adaptation (Ali et al., 2016; Khazaei et al., 2014a), and yield (Ávila et al., 2017; Cruz‐Izquierdo et al., 2012). QTLs controlling abiotic stress responses in faba bean, detected by either QTL mapping or association mapping approaches, have been discussed by Sallam and Ul‐Allah (2019). Given the few QTLs reported in faba bean compared with other pulses, saturation of the genomic regions associated with target regions and QTL validation in multiple environments and genetic backgrounds are needed to uncover reliable marker‐trait associations such as those reported by Aguilar‐Benitez et al. (2020) for pod dehiscence. The marker density in faba bean has recently been significantly increased (Carrillo‐Perdomo et al., 2020; O'Sullivan et al., 2019); this development will facilitate fine QTL mapping and gene identification.

#### 2.3.1 Successful gene discoveries in the absence of a faba bean reference genome

Despite the relatively limited discovery of genes and QTLs for disease resistance and abiotic stress tolerance, the discovery of genes for the seed anti‐nutritional factors v‐c and tannins, which place major limitations on faba bean usage, has progressed considerably very recently. Vicine and convicine are stored in cotyledons of most faba beans at about 1% of dry matter (Khazaei et al., 2019). They are toxic in people who have a hereditary recessive mutation affecting the enzyme glucose‐6‐phosphate dehydrogenase (G6PD, Luzzatto & Arese, 2018). The first two mapping studies on v‐c content (Gutiérrez et al., 2006; Ramsay et al., 1995) revealed that it was controlled by one major locus. Khazaei et al. (2015) showed that the distribution of v‐c concentration was bimodal, which was consistent with the detection of a single major QTL at the previously reported *vc* ^*−*^ locus on faba bean chromosome 1. Later, a robust, breeder‐friendly and high‐throughput KASP marker was developed and validated from this region (Khazaei et al., 2017). This marker was found to reside within the bifunctional riboflavin biosynthesis protein *RIBA1*, the gene for which is now termed *VC1*, that underlies the major v‐c QTL and catalyses a key step in v‐c biosynthesis (Björnsdotter et al., 2020). The *VC1* gene identification, which relied on genetic mapping and gene‐to‐metabolite correlations, now paves the way for development of faba bean cultivars free from v‐c based on new insight into the v‐c biosynthetic pathway.

Seed coat tannins limit faba bean use in food and feed; a low tannin phenotype, characterised by white flower colour, is controlled by two unlinked recessive genes, *zt1* and *zt2*. A comparative mapping approach identified an ortholog of the *M. truncatula* WD40 transcription factor *TTG1* (*Transparent Testa Glabra 1*), located on chromosome 2, as the *zt1* gene (Webb et al., 2016). These results have been recently confirmed by Gutiérrez and Torres (2019), who characterized *zt1* and proved the high similarity of the gene sequence with other legume species. An allele‐specific diagnostic marker was also developed that differentiates *zt1* from other genotypes. Gutiérrez et al. (2020) reported the bHLH transcription factor *VfTT8* (*Transparent Testa8*) located on chromosome 3 as the *zt2* gene. A robust KASP marker for the *zt2* gene is now available (Zanotto et al., 2020).

Besides the successful gene discovery for quality traits mentioned above, progress was also made for gene discovery and development of a diagnostic molecular marker for the terminal inflorescence gene (*ti*) in faba bean (Avila et al., 2006, 2007). The *TFL1* (*Terminal Flower 1*), as main regulator of inflorescence development in legumes (Benlloch et al., 2015), was responsible for the determinate growth habit in faba bean (*Vf_TFL1*) and is located on chromosome 5.

## 3 TRANSCRIPTOMES

A number of transcriptomes have been reported for faba bean (Table 3), albeit in the absence of a reference genome. These datasets were generated from a selection of different genotypes and tissues at various development stages or treatments. Recent reviews of this topic (Maalouf et al., 2019; O'Sullivan & Angra, 2016) described the faba bean transcriptome contributions up to 2016 that were used for the development of molecular markers for genetic mapping (listed in Table 3). Since then, the transcriptome data coverage has been further enriched (Braich et al., 2017; Cooper et al., 2017). A high proportion of transcripts (about 96%) from Webb et al. (2016) was captured by transcriptome data of Braich et al. (2017). The sequence length data were increased at 461 chromosomal loci and provided increased accuracy by Cooper et al. (2017) compared with transcriptome data in Webb et al. (2016). The transcriptome data of Braich et al. (2017) revealed that faba bean, despite its large complex genome, compared similarly with other legume species in expressed gene content.

**TABLE 3:** Summary of published transcriptome data in faba bean

References	Aim of study	Tissue	Output	NGS platforms
Ray and Georges (2010)	Development of EST sequences	Early to mid‐developed embryo	5000 ESTs	454 sequencing technology
Kaur et al. (2012)	Design and evaluation of EST‐SSRs	Young and mature leaf, stem, flower, immature pod, mature pod and immature seed	802 SSRs	454 Roche GS FLX titanium
Kaur, Kimber, et al. (2014)	Development of SNP markers	Leaf	768 SNP markers	Illumina OPA‐bead array
Ray et al. (2015)	Development of NGS libraries to elucidate the v‐c pathway and other genes for the anti‐nutritional factors	5‐ to 6‐days‐old root and etiolated shoot and developing seed coat	8 libraries containing 1.2 million ESTs	454 sequencing
Arun‐Chinnappa and McCurdy (2015)	Generating a genome‐wide transcriptome map of faba bean	Expanding and fully expanded leave, elongating and fully elongated stem, and closed and open flower, whole roots including root hairs, and cotyledon	17,160 unigenes	Illumina HiSeq‐2000
Ocaña et al. (2015)	Transcriptome analysis under ascochyta blight infection	Leaf tissue at 4, 8 and 12 h after inoculation	21,243 transcripts, 39,060 SNPs and 3669 InDels	Illumina
Webb et al. (2016)	SNP discovery	7‐day‐old seedling	653 new mined SNP markers	GS FLX/454 reads
Braich et al. (2017)	Development of reference unigene sets	Immature pod and fully‐open flower	26,295 new transcripts	RNA‐Seq, Illumina HiSeq 2000
Cooper et al. (2017)	Enhancement of faba bean genome resources	Embryos	16,300 unigenes	RNA‐seq, Illumina HiSeq 2500
Alghamdi et al. (2018)	Identify drought stress differentially expressed genes	Root at vegetative and flowering stages	18,327 SSRs	RNA‐seq, Illumina Hiseq 4000
Gao et al. (2020)	Identify response to vernalization genes	Seedling	6852 SSRs in 6552 transcripts	RNA‐seq, Illumina HiSeq 2500
Yang et al. (2020)	Identify salinity stress differentially expressed genes	Seed	4486 differentially expressed genes	RNA‐seq, Illumina Hiseq 4000
Carrillo‐Perdomo et al. (2020)	SNP discovery	Leaf	39,423 transcripts and 105,828 gene‐based SNPs	RNA‐seq, Illumina MiSeq
Björnsdotter et al. (2020)	Uncovering genes associated with the biosynthesis of vicine‐convicine	Young and mature leaf, flower, pod and whole seed at early seed‐filling stage, embryo and pod at mid maturation, and stem	49,277 transcripts	Illumina HiSeq PE150

Next‐generation sequencing (NGS) platforms, especially high‐throughput RNA sequencing (RNA–seq) technology, one of the most powerful tools currently available for transcriptome profiling, has enhanced the efficiency and speed of gene discovery in faba bean (Table 3). For example, the identification and characterization of differential gene expression from tissues subjected to drought (Alghamdi et al., 2018; Wu et al., 2020), vernalization (Gao et al., 2020), and salinity stress (Yang et al., 2020) have benefited greatly. These findings will help in understanding the stress tolerance mechanisms in the crop and will provide resources for functional genomics. Coupled with allelic data and trait mapping, the data will be invaluable in the development of more resilient faba bean varieties. A high‐quality reference transcriptome has been completed (Björnsdotter et al., 2020) and is being expanded to a pan‐transcriptome using data from four different genotypes (Hedin, Hiverna, 153b and 2378), including data from both shoot and root tissues (Escobar‐Herrera et al., 2020). This effort has provided a comprehensive faba bean reference gene set that will be a valuable new resource for differential gene expression analyses and genome annotation.

## 4 CONCLUSIONS AND PERSPECTIVES

Recent technological advances now allow sequencing of the large genome of faba bean. A collaborative reference genome assembly effort is currently underway, coordinated by the NORFAB consortium (Protein for the Northern Hemisphere, https://bit.ly/37QxeuM), and a pan‐genome initiative is being launched by the University of Helsinki and Luke (Natural Resources Institute Finland). The NORFAB project has developed an annotated reference transcriptome for faba bean (Escobar‐Herrera et al., 2020), which will aid the development of gene models for the reference assembly. The transcriptome work has also led to production of a high density faba bean genotyping array, which is now available from the University of Reading, UK. The array (known as ‘Vfaba_v2’), built on Life Technologies Axiom platform, contains 24,929 polymorphic high resolution SNP markers located in 15,846 different genes. Faba bean now benefits from saturated synteny‐based genetic maps, NGS, and high‐throughput genotyping technologies, which together will greatly aid genome assembly. Release of the reference genome will further advance the faba bean genomics and breeding revolution.

## CONFLICT OF INTEREST

The authors have no conflict of interest.

## AUTHOR CONTRIBUTION

H. K. did the writing of the original draft. All authors did the writing, review and editing of the manuscript. All authors read and agreed to the published version of the manuscript.

## ETHICS STATEMENT

This manuscript does not contain any studies with human or animal subjects.

## Data Availability

Data sharing is not applicable to this article as no datasets were generated or analysed during the current study.
